# Phylogenomic analysis of OXA-23-positive and genetic context of rare NDM-1-producing carbapenem-resistant *Acinetobacter baumannii* isolates in a teaching hospital in China

**DOI:** 10.3389/fmicb.2025.1575257

**Published:** 2025-08-06

**Authors:** Su Dong, Jianjiang Lou, Caiping Mao, Yuejuan Fang, Huan Zhang

**Affiliations:** ^1^Department of Clinical Laboratory, Shaoxing Hospital of Traditional Chinese Medicine Affiliated to Zhejiang Chinese Medical University, Zhejiang, China; ^2^Department of Clinical Laboratory, Zhejiang Cancer Hospital, Hangzhou Institute of Medicine (HIM), Chinese Academy of Sciences, Zhejiang, China; ^3^Department of Pharmacy, Quzhou Maternal and Child Health Care Hospital, Quzhou, China

**Keywords:** CRAB, WGS, comparative genomics analysis, SNP, plasmid structure

## Abstract

Nosocomial outbreaks caused by carbapenem-resistant *Acinetobacter baumannii* (CRAB) strains are rapidly emerging worldwide and are a cause for concern. In this study, a phylogenetic tree of 18 *A. baumannii* strains collected from a teaching hospital in China was constructed to explore the genetic relationship in the context of genomic insights. The study also aimed to explore the relationship among the strains and to assess the potential spread within the teaching hospital. All CRAB strains were collected from 17 patients, with the majority obtained from sputum samples (55.56%, 10/18). Moreover, 61.11% (11/18) of the CRAB strains were collected from the intensive care unit (ICU). Whole-genome sequencing (WGS) was performed using the Illumina and Oxford Nanopore platforms, and the bioinformatic analysis was subsequently performed. Based on the Pasteur multilocus sequence typing (MLST) scheme, 16 *A. baumannii* strains were classified as sequence type 2 (ST2). The remaining two *A. baumannii* strains belonged to two rare sequence types (STs), namely ST34 and ST23, respectively. KL and OCL analysis showed that the majority of the strains (61.11%, 11/18) contained KL7. Whole-genome sequencing revealed that 16 CRAB strains were OXA-23 producers, while the remaining two strains carried *bla*_NDM-1_ and *bla*_OXA-72_. Genetic structure analysis showed the context of *bla*_NDM-1_ was IS*Aba14*-*aph(3′)-VI-*IS*Aba125-bla*_NDM-1_-*ble-MBL.* Comparative genomics analysis revealed that 16 CRAB strains (ST2_Pas) had a close genetic relationship, and 8 CRAB strains possessed the same resistance gene profile, with only 1–6 SNPs observed among them. Therefore, there is an urgent need for increased surveillance of both patients and the hospital environment to prevent and control the spread of CRAB.

## 1 Introduction

*Acinetobacter* species, including *Acinetobacter baumannii*, *Acinetobacter calcoaceticus*, *Acinetobacter nosocomialis*, *Acinetobacter pittii*, *Acinetobacter dijkshoorniae*, *Acinetobacter lactucae*, and *Acinetobacter seifertii,* are ubiquitous in diverse clinical settings and environments (Wong et al., 2017; Miller and Arias, 2024; Richards et al., 2024). Among these species, *A. baumannii* is an important opportunistic pathogen that primarily causes healthcare-associated infections, including bloodstream infections (BSIs), pneumonia, and urinary tract infections, especially in immunocompromised patients hospitalized in intensive care units (ICUs) (Harding et al., 2018; Wibberg et al., 2018; Wang S. H. et al., 2024). Carbapenems are usually the first-choice antibiotics recommended for treating serious infections caused by *A. baumannii* (Mathlouthi et al., 2016). However, outbreaks caused by carbapenem-resistant *A. baumannii* (CRAB) strains in hospitals are emerging rapidly worldwide and are a growing concern (Ruan et al., 2013).

The primary mechanism by which *A. baumannii* develops resistance to carbapenems is through the acquisition of genes encoding carbapenem-hydrolyzing class D (*bla*_OXA-23_, *bla*_OXA-24_, and *bla*_OXA-58_) (Doughty et al., 2023) and metallo-*β*-lactamase (MBL) enzymes, including *bla*_NDM-1_, *bla*_IMP_, *bla*_VIM_, and *bla*_GIM_ (Bakour et al., 2015). The most commonly acquired class D carbapenemase gene worldwide is *bla*_OXA-23_ (Capodimonte et al., 2024; Wang W. et al., 2024). Based on previous studies, the *bla*_OXA-23_ gene is usually located in IS*Aba1*-based transposons, namely Tn*2006*, Tn*2008*, Tn*2009*, and the AbaR4-type resistance island (Liu H. et al., 2023). Compared to *bla*_OXA-23_, *bla*_NDM-1_ is relatively rare. However, some studies from Algeria and Nepal have reported the coexistence of *bla*_OXA-23_ and *bla*_NDM-1_ genes (Ramoul et al., 2016; Joshi et al., 2017). Moreover, an increasing proportion of the coexistence of *bla*_OXA-23_ and *bla*_NDM-1_ in CRAB clinical isolates was found in an ICU in Hangzhou, China (Liu et al., 2024). The coexistence of *bla*_OXA-23_ and *bla*_NDM-1_ increases the resistance levels to carbapenems, resulting in limited treatment options (Liu et al., 2024).

In this study, a phylogenetic tree of *A. baumannii* strains collected from a teaching hospital in China was constructed to explore the genetic relationship in the context of genomic insights. This study also aimed to explore the relationship between the strains and assess the potential spread within this hospital. More importantly, the plasmid structure of the *bla*_NDM-1_ gene in a rare clinical CRAB isolate (belonging to ST34_Pas) was further explored. This study provides a basis for better understanding the characteristics of CRAB strains and supports better treatment strategies for OXA-23 or NDM-1-producing isolates.

## 2 Materials and methods

### 2.1 Bacterial isolation and identification

A total of 18 *A. baumannii* strains were collected from a teaching hospital in Hangzhou, Zhejiang, China, during routine diagnostic analysis from 2020 to 2024. All CRAB isolates were collected without applying specific inclusion and exclusion criteria. Isolate identification to the species level was conducted using matrix-assisted laser desorption ionization time-of-flight mass spectrometry (MALDI-TOF MS; Bruker Daltonik GmbH, Bremen, Germany) and confirmed through 16S rRNA sequencing. The sequences of the primers were (27F-5’-AGAGTTTGATCCTGGCTCAG-3′ and 1492R-5’-GGTTACCTTGTTACGACTT-3′).

### 2.2 Antimicrobial susceptibility testing (AST)

Minimum inhibitory concentrations (MICs) of the 18 *A. baumannii* strains were determined using the VITEK 2 compact system with the AST-N335 card. The results were interpreted according to the recommendations outlined in the Clinical and Laboratory Standards Institute (CLSI) 2021 guidelines. The following antimicrobial agents were investigated in this study: imipenem (IPM), meropenem (MEM), ceftazidime (CAZ), ciprofloxacin (CIP), levofloxacin (LEV), tobramycin (TOB), and polymyxin (PB). In addition, the MIC of cefiderocol (CFDC) was determined using the broth microdilution method in iron-depleted cation-adjusted Mueller–Hinton broth (ID-CAMHB) according to the CLSI guidelines (Liu X. et al., 2023).

### 2.3 Mating experiments

To determine whether the plasmid carrying *bla*_NDM-1_ was transferable, conjugation experiments using *E. coli* J53 (Sodium azide resistant) as the recipient strain were carried out using the film mating method (Yang et al., 2021). Transconjugants were screened on Mueller–Hinton (MH) agar plates containing sodium azide (100 mg/L) and meropenem (4 mg/L), and the putative transconjugants were confirmed via PCR and MALDI-TOF MS. The putative transconjugants were first identified as *E. coli* using MALDI-TOF MS and then confirmed to carry the *bla*_NDM-1_ gene through PCR (Tian et al., 2023).

### 2.4 Whole-genome sequencing (WGS)

#### 2.4.1 Illumina sequencing

Genomic DNA was extracted using the Qiagen Mini Kit (Qiagen; Hilden, Germany) following the manufacturer’s instructions. In brief, one colony from each purified culture was cultured in 2 mL MH broth for 24 h at 37°C. Cell pellets were harvested and added to 180 μL of Buffer ATL containing 20 μL of Proteinase K (Qiagen, Germany), followed by incubation for 2 h. Then, 200 μL of Buffer AL was added, and the mixture was incubated at 70°C for 10 min. The mixture was transferred to a QIAamp Mini spin column and centrifuged at 12,000 × g for 30 s. Then, the column was washed with Buffer AW1 and AW2. DNA was eluted with distilled water by centrifugation at 10,000 × g for 30 s. The quality and quantity of the DNA were assessed using a NanoDrop 2000 spectrophotometer (Thermo Scientific, United States) and a Qubit 4.0 fluorometer (Invitrogen, United States). Illumina sequencing libraries were prepared using the TruePrepTM DNA Library Prep Kit V2 (Vazyme) following the manufacturer’s protocol. Individual libraries were assessed using the QIAxcel Advanced automatic nucleic acid analyzer with a high-resolution gel cartridge (Qiagen, Germany) and were quantified by qPCR using the KAPA SYBR FAST qPCR Kit (Kapa KK4610, KAPA Biosystem, Wilmington, MA, United States). Paired-end sequencing (2 × 150-bp reads) was performed on the Illumina HiSeq X Ten platform (Illumina Inc., San Diego, CA, United States).

#### 2.4.2 Oxford Nanopore sequencing

Long-read sequencing was performed for two rare clinical CRAB isolates (belonging to ST34_Pas and ST23_Pas), which harbor *bla*_NDM-1_ and *bla*_OXA-72_ genes. For this process, DNA was extracted using the Gentra^®^ Puregene^®^ Yeast/Bact Kit (Qiagen, Germany) following the manufacturer’s protocol, with minor modifications. Oxford Nanopore sequencing libraries were prepared using the SQU-LSK109 Ligation Sequencing Kit (Oxford Nanopore Technologies, UK) in conjunction with the PCR-Free ONT EXP-NBD104 Native Barcode Expansion Kit (Oxford Nanopore Technologies, UK), following the native barcoding genomic DNA protocol. The DNA was processed without the optional shearing steps to select for long reads. After quantification of the individual libraries using the Qubit fluorometer and normalization of library concentrations, the library was sequenced on the GridION X5 platform (Oxford Nanopore Technologies, UK).

### 2.5 Bioinformatic analyses of the genomes

Genome assemblies of the short reads of Illumina were performed using the Shovill (version 1.0.9) pipeline. Assemblies of the short and long reads of Illumina and MinION were generated using Unicycler v0.4.8 (Wick et al., 2017). The quality of the 18 genome sequences was assessed using QUAST v5.0.2 (Gurevich et al., 2013). The quality results of the 18 assembly sequences are shown in Supplementary Table S1 (all N50 > 100 kb). Genome sequence prediction and annotation were performed using Prokka 1.11 (Seemann, 2014) and the National Center for Biotechnology Information (NCBI) Prokaryotic Genome Annotation Pipeline (PGAP)^1^ (Tatusova et al., 2016). Antimicrobial resistance genes were identified using the ABRicate program^2^ according to the ResFinder database. Multilocus sequence typing (MLST) using both the Oxford and Pasteur schemes was performed via the Center for Genomic Epidemiology (CGE) website^3^. Bacterial virulence factors were identified using the Virulence Factor Database (VFDB)^4^ (Liu et al., 2022). Capsular polysaccharide (K locus) and lipoolygosaccharide (OC locus) were further identified using Bautype and *Kaptive* v2.0.0 (Lam et al., 2022). Sequence comparisons were performed using BLASTn v2.4.0 (Zhang et al., 2000). Plasmid structure was visualized using DNAplotter.^5^ The origin-of-transfer (*oriT*) region and horizontal transfer of bacterial mobile genetic elements (MGEs) for plasmids were predicted using oriTDB^6^ (Liu et al., 2025). Similar plasmids were tracked using BacWGSTdb^7^ (Ruan and Feng, 2016). Default parameters were used for all software.

### 2.6 Comparative genomics analysis

Comparative genomics analysis of the 18 A. baumannii strains was performed. In detail, Snippy v4.4.5^8^ was utilized to align the Illumina reads against the reference strain (A. baumannii ATCC 19606, accession number: GCF_900011295.1) and to generate a core genome alignment (approximately 4,000,000 bp), with repetitive regions removed using Gubbins v2.4.1 (Croucher et al., 2015). Final phylogenies were constructed using IQ-TREE v2.0.3 (Croucher et al., 2015; Nguyen et al., 2015). The resulting tree file was further visualized with the Interactive Tree of Life (iTOL v5) tool. The alignments were used to calculate SNP distances with SNP-dists V0.6.3^9^ and visualized by RStudio version 3.5.3. Default parameters were used for all software.

## 3 Results

### 3.1 Collection of CRAB isolates and patients’ information

In this study, a total of 18 CRAB strains were collected from 17 patients (11 male and six female individuals; age range: 52–80 years) at a teaching hospital from 2020 to 2024. These strains were primarily collected from sputum samples (55.56%, 10/18), followed by secretions (16.67%, 3/18). Moreover, 61.11% (11/18) of the CRAB strains were collected from the ICU, while 16.67% (3/11) were collected from the gastric surgery department (Table 1). The remaining four strains were isolated from the urology, hematology, general internal medicine, and colorectal surgery departments, respectively (Table 1). The primary diagnosis among the 17 patients was tumor (72.22%, 13/18). Detailed information is provided in Table 1.

**Table 1:** Information on the 18 clinical CRAB strains included in this study.

Isolate ID	Collection date	Patient ID	Sex	Age	Admission date	Discharge date	Sample type	Clinical department	Diagnosis
SZL6	24.01.13	P1	Female	73	23.12.25	24.01.15	Sputum	Gastric surgery	Tumor
SZL8	24.03.11	P2	Male	71	24.03.04	24.04.05	Sputum	ICU	Tumor
SZL9	24.03.25	P3	Female	52	24.03.20	24.04.12	Sputum	ICU	Tumor
SZL10	24.03.20	P4	Male	53	24.03.16	24.03.22	Sputum	ICU	Cerebral hemorrhage
SZL11	24.04.09	P5	Female	52	24.03.20	24.04.12	Secretions	ICU	Tumor
SZL13	24.03.27	P6	Female	73	24.01.19	24.04.17	Sputum	ICU	Liver disease
SZL14	23.05.30	P7	Male	57	23.05.24	23.06.07	Urine	Urology	Tumor
SZL15	24.05.03	P8	Male	72	24.05.03	24.05.05	Throat swab	Hematology	Diffuse large B-cell lymphoma
SZL17	24.06.17	P9	Male	76	24.06.06	24.06.21	Sputum	General internal medicine	Respiratory failure
SZL18	24.06.15	P10	Male	80	24.05.28	24.07.01	Sputum	Gastric surgery	Tumor
SZL19	24.07.01	P11	Male	72	24.06.03	24.07.03	Drainage fluid	ICU	Tumor
SZL20	23.08.02	P12	Male	69	23.07.27	23.08.07	Sputum	Colorectal surgery	Tumor
SZL21	24.07.09	P13	Male	70	24.05.31	24.08.05	Sputum	Gastric surgery	Tumor
SZL24	23.10.09	P14	Male	73	23.06.28	24.04.15	Sputum	ICU	Tumor
SZL25	23.10.18	P15	Male	67	23.10.10	23.10.22	Secretions	ICU	Tumor
SZL29	20.08.19	P16	Male	68	20.08.10	20.08.19	Drainage fluid	ICU	Septic shock
SZL191	20.06.24	P17	Female	65	20.06.24	20.07.14	Secretions	ICU	Tumor
SZL194	23.06.30	P17	Female	65	20.06.24	20.07.14	Blood	ICU	Tumor

### 3.2 Drug resistance profiles

Antimicrobial susceptibility testing (AST) revealed that the 18 *A. baumannii* strains exhibited resistance to IMP and MEM (Table 2). Apart from SZL20, all other strains were resistant to CAZ, and 88.89% (16/18) of the strains were found to be resistant to CIP. Moreover, 83.33% (15/18) and 72.22% (13/18) of the strains were resistant to LEV and TOB, respectively (Table 2). In addition, two strains were observed to be resistant to PB, with MIC values of 4 and 8, respectively. Importantly, all *A. baumannii* strains were susceptible to CFDC, with MICs of 2 μg/mL or 4 μg/mL.

**Table 2:** MLST, KL, OCL, and MICs for the 18 *A. baumannii* clinical strains.

Strains	ST (pas)	ST (oxf)	KL	OCL	MICs (μg/mL)							
					PB	CIP	MEM	IPM	LEV	CAZ	TOB	CFDC
SZL6	2	938	KL93	OCL1c	≤0.5	≥4	≥16	≥16	4	≥64	≥16	2
SZL8	2	208	KL7	OCL1	≤0.5	≥4	≥16	≥16	≥8	≥64	≥16	2
SZL9	2	208	KL7	OCL1	≤0.5	≥4	≥16	≥16	≥8	≥64	≥16	2
SZL10	2	208	KL7	OCL1	4	≥4	≥16	≥16	≥8	≥64	≥16	2
SZL11	2	208	KL7	OCL1d	≤0.5	≥4	≥16	≥16	≥8	≥64	≥16	2
SZL13	2	208	KL7	OCL1	≤0.5	≥4	≥16	≥16	≥8	≥64	≥16	2
SZL14	2	208	KL7	OCL1	8	1	≥16	≥16	0.5	≥64	≥16	2
SZL15	34	432	KL119	OCL2	≤0.5	≥4	≥16	≥16	≥8	≥64	≥16	4
SZL17	2	195	KL3	OCL1	≤0.5	≥4	≥16	≥16	≥8	≥64	≥16	2
SZL18	2	208	KL7	OCL1c	≤0.5	≥4	≥16	≥16	≥8	≥64	≥16	4
SZL19	2	208	KL7	OCL1	≤0.5	≥4	≥16	≥16	≥8	≥64	≥16	2
SZL20	23	642	KL108	OCL2	≤0.5	≤0.25	≥16	8	≤0.12	2	≤1	2
SZL21	2	469	KL45	OCL1c	2	≥4	≥16	≥16	≥8	≥64	≥16	2
SZL24	2	208	KL2	OCL1	≤0.5	≥4	≥16	≥16	≥8	≥64	≥16	2
SZL25	2	540	KL22	OCL1c	≤0.5	≥4	≥16	≥16	≥8	≥64	≥16	4
SZL29	2	208	KL7	OCL1d	≤0.5	≥4	≥16	≥16	≥8	≥64	≤1	2
SZL191	2	208	KL7	OCL1d	≤0.5	≥4	≥16	≥16	≥8	≥64	≤1	2
SZL194	2	208	KL7	OCL1d	≤0.5	≥4	≥16	≥16	≥8	≥64	≤1	2

PB: Polymyxin; CIP: levofloxacin; IMP: Imipenem; MEM: Meropenem; LEV: Levofloxacin; CAZ: Ceftazidime; TOB: Tobramycin; CFDC: cefiderocol.

### 3.3 Virulence genes

Many virulence factors were identified in our CRAB strains, including the *pga* operon (*pgaABCD*), which encodes poly-*β*-1,6-N-acetyl-d-glucosamine (PNAG), and the outer membrane protein gene *ompA*. In addition, the *csu* operon encoding Csu pili, the two-component regulatory system *bfmRS*, and other genes (*bauABCDEF*, *basABCDEFGHIJ,* and *barAB*) encoding acinetobactin for iron uptake were also identified in these strains.

### 3.4 Multilocus sequence typing (MLST), KL, and OCL

Based on the Pasteur MLST scheme, 16 *A. baumannii* strains were classified as sequence type 2 (ST2). The remaining two *A. baumannii* strains belonged to two rare sequence types (STs), namely ST34 and ST23, respectively (Table 2). Based on the Oxford MLST scheme, 12 *A. baumannii* strains belonged to ST208, while the remaining strains were assigned to ST938, ST432, ST195, ST642, ST469, and ST540, respectively (Table 2).

KL and OCL analysis showed that the majority of the strains (61.11%, 11/18) contained KL7. Concerning OCL types, OCL1, OCL1c, and OCL1d were identified, collectively accounting for 88.89% (16/18). The remaining two *A. baumannii* strains were classified as OCL2 (Table 2).

### 3.5 Resistance gene distribution and comparative genomics analysis of the *A. baumannii* strains

The analysis of the 18 CRAB genomes in this study revealed that, in addition to co-harboring chromosomal *ant(3″)-IIa*, many resistance genes conferring resistance to different types of antimicrobial agents were identified. Moreover, 16 CRAB strains were OXA-23 producers, and two strains were found to carry *bla*_NDM-1_ and *bla*_OXA-72_. In addition, 72.22% (13/18) of the strains were found to harbor the ribosome-methylase encoding gene *armA*. A total of 13 CRAB isolates were all resistant to TOB.

**Figure 1:**
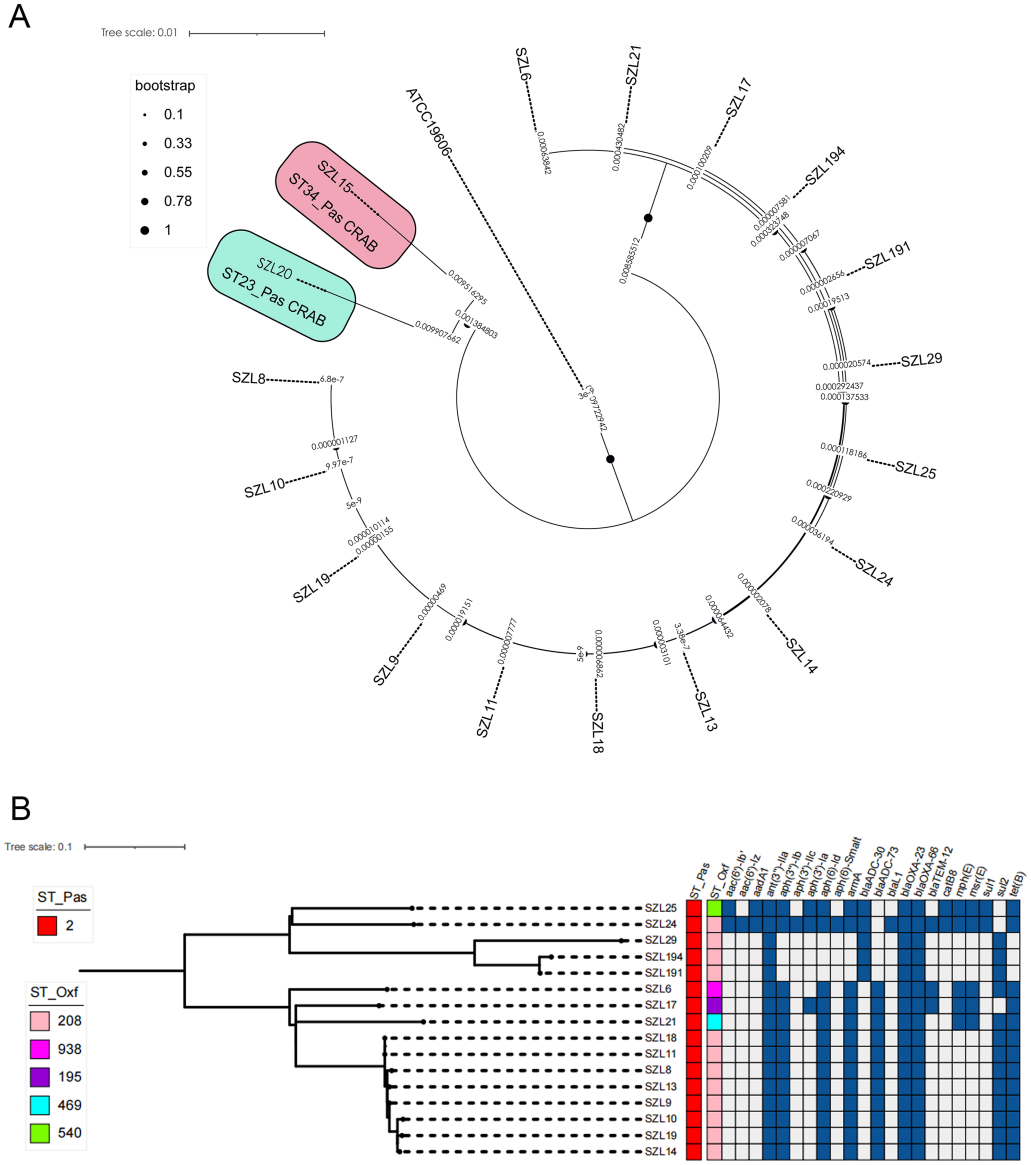
Phylogenetic analysis of the 18 *A. baumannii* isolates. **(A)** Phylogenetic analysis was performed using the reference strain (*A. baumannii* ATCC 19606, accession number: GCF_900011295.1). Snippy v4.4.5 was utilized to align the Illumina reads against the reference strain and to generate a core genome alignment, with repetitive regions removed using Gubbins v2.4.1. Final phylogenies were constructed using IQ-TREE (version 2.0.3). Special STs (ST23 and ST34) are labelled, while the remaining strains are all ST2 CRAB strains. **(B)** Phylogenetic analysis was performed using IQ-TREE (version 2.0.3). The tree was visualized using iTOL v5. Isolate names, ST_Pasteur, ST_Oxford, and resistance genes are shown for each isolate.

**Figure 2:**
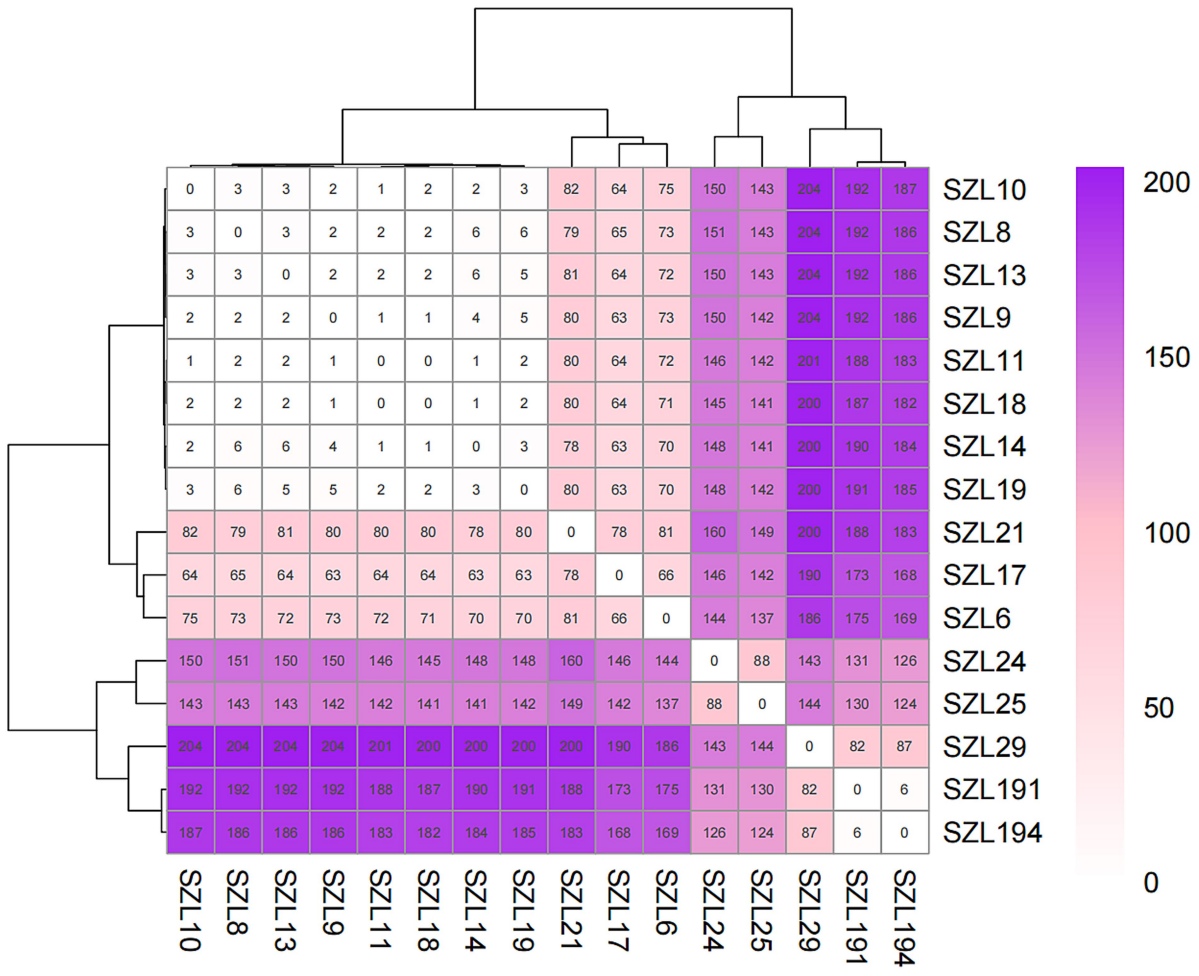
SNP matrix values for the ST2_Pasteur *A. baumannii* strains. SNP differences are indicated as numbers within the boxes.

To explore the genetic relationship among the *A. baumannii* strains, comparative genomics analysis was performed on the 18 CRAB strains. The result showed that 16 CRAB strains (ST2_Pas) had a relatively close genetic relationship, which was consistent with the results of MLST (Figure 1A). We further constructed a tree of the 16 CRAB strains. The results revealed that SZL8, SZL9, SZL10, SZL10, SZL11, SZL13, SZL14, SZL18, and SZL19 had a near genetic relationship within our hospital (Figure 1B). Clinical data revealed that six of the eight strains were isolated from the ICU, suggesting possible transmission within the ICU of the teaching hospital. The eight CRAB clinical isolates shared the same resistance gene profile. Apart from SZL14, all other CRAB isolates were collected in 2024. The heatmap of the resistance genes is shown in Figure 1B. SNP analysis showed that only 1–6 SNPs were observed among these strains (Figure 2). In addition, SZL191 and SZL194 also had a quite close genetic relationship, and they were collected from the same patient (P17).

### 3.6 Genetic analysis of the chromosome and plasmids in the rare ST34 CRAB

The hybrid assembly of the Illumina and MinION reads showed that the *A. baumannii* SZL15 strain possessed a circular chromosome of 3,703,254 bp (Table 3). A total of three plasmids were identified in this strain, namely pSZL15-1 to pSZL15-3, with sizes ranging between 8,692 bp and 99,894 bp. pSZL15-2 carried different kinds of resistance genes, and *bla*_NDM-1_ was identified in this plasmid. However, no resistance genes were found in pSZL15-1 and pSZL15-3 (Table 3).

**Table 3:** Molecular characterization of the genome of the rare ST34 *A. baumannii* clinical strains.

Genome	Size (bp)	Resistance genes	Accession number
Chromosome	3,703,254	*ant(3″)-IIa*, *bla*_ADC-123_, *bla*_OXA-430_	CP176840
pSZL15-1	99,894	ND	CP176841
pSZL15-2	48,557	*aph(3′)-VI*, *bla*_NDM-1_, *ble-MBL*	CP176842
pSZL15-3	8,692	ND	CP176843

ND: not detected.

### 3.7 Genetic features of the NDM-1-positive plasmid pSZL15-2 and identification of similar plasmids in the public database

**Figure 3:**
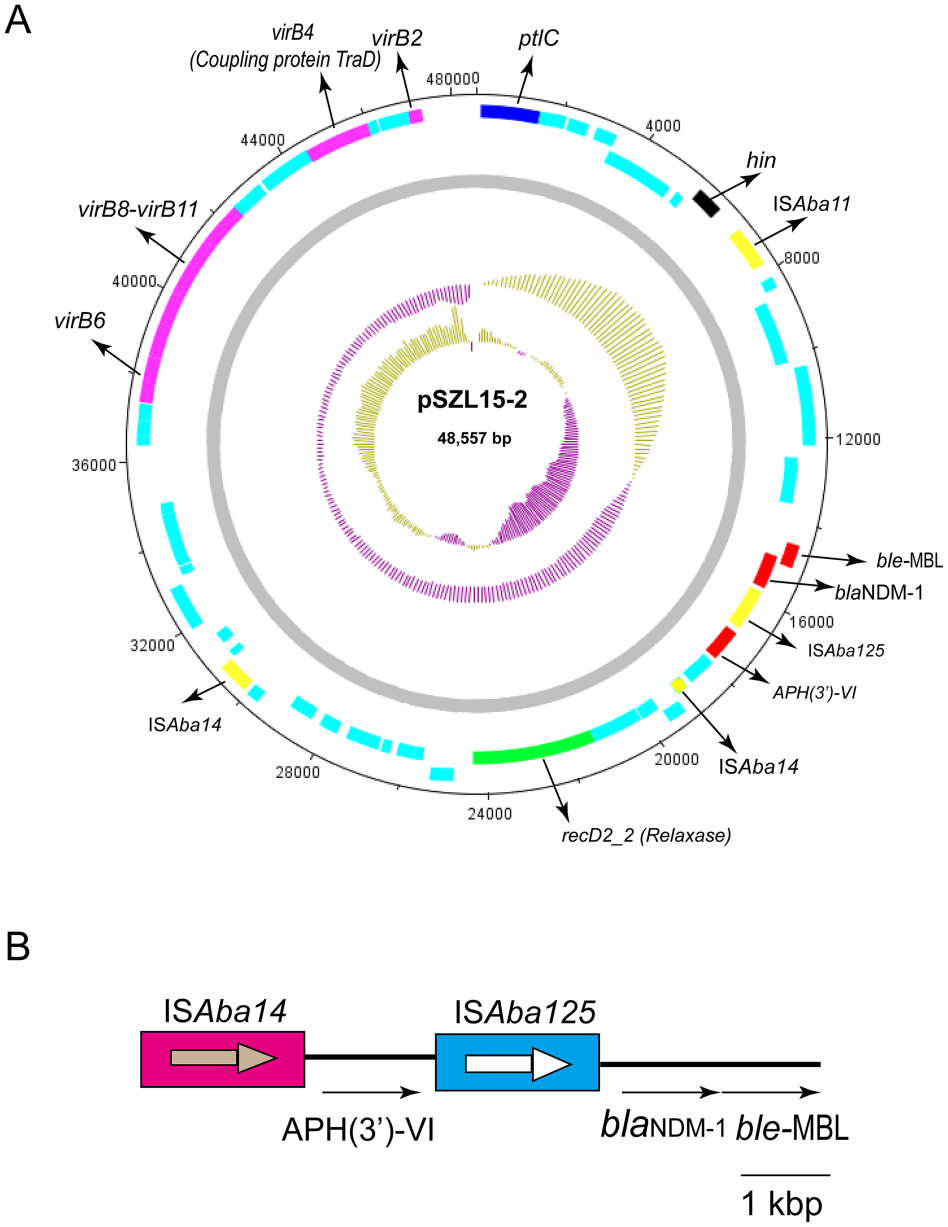
Circular map of pSZL15-2 and the genetic structure of *bla*_NDM-1_. **(A)** Circular map of the pSZL15-2 plasmid. IS copies are shown as yellow-filled boxes. Resistance genes are shown as red-filled boxes. T4SS-related genes, including *virB*, coupling protein, and relaxase encoding genes, are shown. Light blue-filled boxes represent other ORFs. **(B)** Structure of the *bla*_NDM-1_ gene.

Genetic analysis revealed that the *bla*_NDM-1_ gene cluster was arranged sequentially as IS*Aba125*, *bla*_NDM-1_, and *ble-MBL* elements (Figure 3). However, the upstream of IS*Aba125* was the genetic context of IS*Aba14-aph(3′)-VI* (Figure 3). The *bla*_NDM-1_ gene was embedded within a Tn*6924*-like composite transposon. Mating assays were performed to explore the transfer ability of the *bla*_NDM-1_ gene, and the results showed that the *bla*_NDM-1_-harboring plasmid could not be transferred to the recipient strain. The result of oriTDB2 showed that no *oriT* was found in the *bla*_NDM-1_-harboring plasmid. Comparison with similar plasmids in public databases showed that *bla_NDM-1_*-positive plasmids share high sequence identity with plasmids identified in other *Acinetobacter* sp. and *A. baumannii* strains collected from different countries (Table 4). These plasmids were isolated from both patient and animal sources, suggesting potential horizontal transfer of *bla*_NDM-1_-positive plasmids across hosts.

**Table 4:** Information on similar plasmids from the public database.

Accession number	Plasmid	Strain	Identity	Size	Species	Host	Source	Location	Year	Resistance genes
KM210088.1	pNDM-JN02	JN247	0.999952	41,084	*Acinetobacter* sp.	*Homo sapiens*	Feces	China	2011	*aph(3′)-VI*, *bla*_NDM-1_
NC_025116.1	pM131_NDM1	M131	0.999905	47,271	*Acinetobacter* sp.	-	-	-	-	*aph(3′)-VI*, *bla*_NDM-1_
NZ_CP032278.1	pNDM1_010034	WCHAc010034	0.999618	49,649	*Acinetobacter* sp.	-	Hospital sewage	China	2015	*aph(3′)-VI*, *bla*_NDM-1_
MT002974.1	pAB17	AB17	0.999952	41,087	*A. baumannii*	*Homo sapiens*	-	Brazil	-	*aph(3′)-VI*, *bla*_NDM-1_
NZ_CP027532.1	Unnamed2	AR_0088	0.999952	41,087	*A. baumannii*	-	-	-	-	*aph(3′)-VI*, *bla*_NDM-1_
NZ_CP010399.1	p6200-47.274 kb	6,200	0.999809	47,274	*A. baumannii*	*Homo sapiens*	Bodily fluid	Colombia	2012	*aph(3′)-VI*, *bla*_NDM-1_
NC_019985.2	pAbNDM-1	ZW85-1	0.999761	48,368	*A. baumannii*	-	-	-	-	*aph(3′)-VI*, *bla*_NDM-1_
NC_019268.1	pNDM-BJ01	WJ10621	0.999809	47,274	*A. lwoffii*	-	-	-	-	*aph(3′)-VI*, *bla*_NDM-1_
NC_019281.1	pNDM-BJ02	WJ10659	0.999952	46,165	*A. lwoffii*	-	-	-	-	*aph(3′)-VI*, *bla*_NDM-1_
NC_025000.1	pNDM-Iz4b	Iz4b	0.999761	46,570	*A. lwoffii*	*Homo sapiens*	-	China	-	*aph(3′)-VI*, *bla*_NDM-1_
NZ_CP041229.1	pAhaeAN54e	AN54	0.999952	45,460	*A. haemolyticus*	*Homo sapiens*	Peritoneal dialysis fluid	Mexico	2016	*aph(3′)-VI*, *bla*_NDM-1_
AMXH01000087.1	pXM1	XM1570	0.999809	47,274	*A. pittii*	*Homo sapiens*	Sputum	China	2010	*aph(3′)-VI*, *bla*_NDM-1_
LC483156.1	pSU1805NDM	SU1805	0.999857	41,022	*A. pittii*	-	Hospital environment	Japan	-	*aph(3′)-VI*, *bla*_NDM-1_
MK053934.1	pIEC38057	IEC38057	0.999857	41,085	*A. nosocomialis*	-	Blood	Brazil	2016	*aph(3′)-VI*, *bla*_NDM-1_
NZ_CP010370.2	p6411-9.012 kb	6,411	0.999809	47,274	*A. nosocomialis*	*Homo sapiens*	Excreted bodily substance	Colombia	2012	*aph(3′)-VI*, *bla*_NDM-1_
NC_023322.1	pNDM-40-1	CHI-40-1	0.999761	45,826	*A. bereziniae*	-	-	-	-	*aph(3′)-VI*, *bla*_NDM-1_
NZ_CP045130.1	p4TQ-NDM	TQ04	0.999713	41,086	*A. indicus*	Cow	Cow feces	China	2017	*aph(3′)-VI*, *bla*_NDM-1_
NZ_CP045197.1	p23TQ-NDM	TQ23	0.999618	41,393	*A. indicus*	Cows	Cow feces	China	2017	*aph(3′)-VI*, *bla*_NDM-1_
NZ_CP035935.1	pNDM1_060092	WCHAc060092	0.999618	48,560	*A. cumulans*	-	Sewage	China	2018	*aph(3′)-VI*, *bla*_NDM-1_

Different colors represent various species.

## 4 Discussion

*A. baumannii* is a nosocomial pathogen that causes ventilator-associated infections and bloodstream infections (BSIs) in critically ill patients, and the spread of CRAB strains is of great concern (Harding et al., 2018). More importantly, CRAB can disseminate resistance rapidly via horizontal gene transfer (HGT) among various strains from environmental and patient sources, especially in the ICU (Zhao et al., 2022). Previous reports have shown that resistance to carbapenems is usually mediated by OXA-23, OXA-24, and OXA-58-type carbapenemases in *A. baumanni* (Mentasti et al., 2020). Consistent with previous studies, the production of the OXA-23 carbapenemase was the main resistance mechanism in this study (Liu et al., 2024). Further studies concerning the genetic environment of *bla*_OXA-23_ (Tn*2006* or Tn*2009* transposon) could be performed. Moreover, we found that the ICU is a crucial location for the development and spread of CRAB strains. In a recent report, Liu *et al*. found that 71.4% (55/77) of the ICUs were contaminated by CRAB strains, which is consistent with our findings (Doughty et al., 2023).

Apart from OXA-23-mediated carbapenem resistance, MBL-mediated resistance, such as NDM-1-mediated carbapenem resistance, was also observed in one rare CRAB strain. *A. baumannii* strains carrying *bla*_NDM-1_ have been reported in clinical and environmental isolates across several countries (Bontron et al., 2016; Jaidane et al., 2018). Notably, the most common *A. baumannii* strains harboring *bla*_NDM-1_ usually belonged to ST85 and ST25 in previous studies, which is distinct from our strain (Bakour et al., 2014; Rafei et al., 2015; Ramoul et al., 2016). Here, the *bla*_NDM-1_ gene was identified in a rare ST34 CRAB strain based on the Pasteur scheme. Previous studies have shown that plasmids containing conjugative elements, including the origin-of-transfer (*oriT*) region, relaxase enzyme, type IV coupling protein (T4CP) gene, and a gene cluster encoding the type IV secretion system (T4SS), are capable of transfer (Liu et al., 2025). The *oriT* region is essential for the transfer process, as it is recognized by the relaxase enzyme and undergoes nicking at a conserved site (nic), resulting in the formation of single-stranded DNA (ssDNA) (Liu et al., 2025). However, no *oriT* region was found in our plasmid, which likely explains its lack of transferability.

Based on genetic structure analysis, we found that the *bla*_NDM-1_ gene was located within a 76-kb Tn*6924*-like composite transposon. Tn*6924* and Tn*6924*-like transposons are novel members of the Tn*7* family with IS*Aba14* and some resistance genes such as *bla*_NDM_ and *aph(3′)-VI* (*aphA6*) (Mann et al., 2022). The Tn*125* composite transposon contains two copies of IS*Aba125* with 3-bp target site duplications (TSDs). However, no IS*Aba14* was identified in the Tn*125* composite transposon. Poirel *et al*. found that *bla*_NDM-1_ was located within the 10,099-bp composite transposon Tn*125,* bracketed by two copies of IS*Aba125* (Poirel et al., 2012). Tn*125* seems to be the primary vehicle for the spread of *bla*_NDM-1_ in *A. baumannii*. Another study from China also identified the structure of Tn*125* in *A. baumannii* strains (Liu et al., 2021), which differs from our research.

The OCL and KL gene clusters, which are responsible for the biosynthesis of the OCL and capsule, respectively, serve as potentially useful epidemiological markers (Tian et al., 2022). They may play an important role in vaccine development (Tian et al., 2022). In the current study, we identified KL7 and OCL1 as the important types; vaccines targeting these types could be explored in the future. Previous studies have shown that ST208 (Oxford scheme) and KL7 isolates exhibit higher virulence and biofilm formation ability, with KL7 *A. baumannii* isolates also showing higher capsule production (Chen et al., 2024). Another finding was that the majority of the isolates belonged to ST2 based on the Pasteur MLST scheme. In a previous study, researchers found that OXA-23-producing global clone 2 (GC2) isolates dominated (99.3%) in an ICU in Zhejiang, China (Doughty et al., 2023). They confirmed that all OXA-23-producing isolates were resistant to imipenem, meropenem, and ciprofloxacin but remained sensitive to polymyxin B and tigecycline. Therefore, polymyxin B and tigecycline can be effective treatment options for OXA-23-positive CRAB isolates. Moreover, cefiderocol (FDC; S-649266), a novel siderophore cephalosporin, has been proven to possess a broad activity against CRAB *in vitro* and *in vivo* (Karruli et al., 2023). However, a study from Spain reported that cefiderocol-resistant strains were related to NDM-1 carbapenemase production and mutations in AmpC. Therefore, it is necessary to monitor NDM-1-producing CRAB isolates in clinical settings to avoid treatment failure due to inappropriate antimicrobial choices.

In addition, a multi-country cohort study showed that 49 (77%) of 64 strains from Fiji and all 32 (100%) strains from Samoa belonged to ST2 CRAB (Baleivanualala et al., 2023; Baleivanualala et al., 2024). These results highlight the global importance of ST2 CRAB and underscore the urgent need for more effective measures to prevent further clonal spread. Furthermore, based on SNP differences, we also obtained evidence of clonal strain transmission in this hospital via core-genome (cg) SNP-based phylogenetic tree and SNP analyses. The clonal spread may have been facilitated by contact between healthcare workers and contaminated environmental surfaces within the hospital. Effective infection control measures, such as implementing “water-free” cleaning methods for patients, ceasing the operation of sinks, increasing the frequency of cleaning hospital device surfaces, and enforcing hand hygiene practices among staffs (Liu et al., 2024), need to be implemented in hospitals to reduce the risk of further CRAB spread, especially in the ICU. Moreover, new drugs such as cefiderocol and sulbactam-durlobactam have demonstrated effective therapeutic capabilities and should be considered promising treatment options for infections caused by CRAB.

However, some limitations remain in this study. The virulence level was not assessed using an insect larvae model or murine infection model. In addition, this was a single-center, observational, retrospective study with a small sample size. We need to collect more CRAB isolates from multiple hospitals, as well as all global CRAB genomes, for a comprehensive comparative genomics analysis. In addition, further investigations are needed to determine whether *bla*_NDM-1_-harboring resistance plasmids could be transferred to other recipient bacteria via conjugation or transformation.

In conclusion, this genome-based study of clinical CRAB isolates collected from a teaching hospital in China between 2020 and 2024 revealed their epidemiological and genomic characteristics.

The OXA-type carbapenemase-encoding gene (*bla*_OXA-23_) was the major determinant of carbapenem resistance. Clone spread has occurred within this hospital. Therefore, it is urgent to implement a routine screening procedure to monitor the presence of CRAB among patients admitted to hospital units, such as ICUs, to prevent and control the spread of CRAB in high-risk patients.

## Data Availability

The datasets presented in this study can be found in online repositories. The names of the repository/repositories and accession number(s) can be found at: https://www.ncbi.nlm.nih.gov/genbank/, PRJNA1202876.

## Footnotes

^1^ http://www.ncbi.nlm.nih.gov/genome/annotation_prok/

^2^ https://github.com/tseemann/abricate

^3^ https://cge.food.dtu.dk/services/MLST/

^4^ http://www.mgc.ac.cn/VFs/

^5^ https://www.sanger.ac.uk/tool/dnaplotter/

^6^ https://bioinfo-mml.sjtu.edu.cn/oriTDB2/

^7^ http://bacdb.cn/BacWGSTdb/index.php

^8^ https://github.com/tseemann/snippy

^9^ https://github.com/tseemann/snp-dists
